# The associations between late effects of cancer treatment, work ability and job resources: a systematic review

**DOI:** 10.1007/s00420-020-01567-w

**Published:** 2020-09-15

**Authors:** Ingrid G. Boelhouwer, Willemijn Vermeer, Tinka van Vuuren

**Affiliations:** 1 Department of Applied Psychology, Amsterdam University of Applied Sciences, Wibauthuis, Wibautstraat 3b, 1091 GH Amsterdam, The Netherlands; 2 Faculty of Management, Open University of The Netherlands, Heerlen, The Netherlands; 3 Loyalis Knowledge and Consult, Heerlen, The Netherlands

**Keywords:** Cancer treatment, Job resources, Late effects, Work ability, Work ability index

## Abstract

**Objective:**

The aim of this review is to evaluate associations between possible late effects of cancer treatment (i.e. physical complaints, fatigue, or cognitive complaints) and work ability among workers beyond 2 years after cancer diagnosis who returned to work. The role of job resources (social support, autonomy, leadership style, coaching, and organizational culture) is also evaluated.

**Methods:**

The search for studies was conducted in PsycINFO, Medline, Business Source Premier, ABI/Inform, CINAHL, Cochrane Library and Web of Science. A quality assessment was used to clarify the quality across studies.

**Results:**

The searches included 2303 records. Finally, 36 studies were included. Work ability seemed to decline shortly after cancer treatment and recover in the first 2 years after diagnosis, although it might still be lower than among healthy workers. No data were available on the course of work ability beyond the first 2 years. Late physical complaints, fatigue and cognitive complaints were negatively related with work ability across all relevant studies. Furthermore, social support and autonomy were associated with higher work ability, but no data were available on a possible buffering effect of these job resources on the relationship between late effects and work ability. As far as reported, most research was carried out among salaried workers.

**Conclusion:**

It is unknown if late effects of cancer treatment diminish work ability beyond two years after being diagnosed with cancer. Therefore, more longitudinal research into the associations between possible late effects of cancer treatment and work ability needs to be carried out. Moreover, research is needed on the buffering effect of job resources, both for salaried and self-employed workers.

**Electronic supplementary material:**

The online version of this article (10.1007/s00420-020-01567-w) contains supplementary material, which is available to authorized users.

## Introduction

A growing number of people in the workforce have experienced a cancer diagnosis at some time during their life. The majority of working people diagnosed with cancer re-enter the workplace. The mean rates of return to work reported in reviews are 62% (Spelten et al. 2002), 64% (Mehnert 2011), and 73% (De Boer et al. 2020a). Return to work pathways vary, among others because of differences in reintegration strategies between countries (Kiasuwa Mbengi et al. 2018), the availability of disability pension (Tikka et al. 2017), or the effectiveness of programs to support return to work (Boer et al., 2015). 

Compared to healthy people 1.4 times more unemployment is observed among cancer patients (De Boer et al. 2009). However, the group of workers with a cancer diagnosis in their life history will continue to expand as survival rates are greatly improving, as the incidence of cancer is expected to rise a further 75% over the next two decades (World Health Organization 2012; Stewart and Wild 2014) and as the retirement age is expected to be raised even further in many countries. As studies concerning cancer and work merely focus on the first two years after diagnosis and often concern whether people return to work, less is known about the population after return to work beyond these first two years. As a consequence, it is important to focus on the occupational well-being and the situation in the workplace of this group of workers after they returned to work. 

A range of long-term physical and psychological changes can be experienced by cancer survivors (Ganz 2001). These changes may present during active treatment and persist on the long term, beyond the first two years after cancer diagnosis, or changes may appear months or years later as late effects (Stein et al. 2008). As a clear distinction between long-term and late effects is not always possible, in this review all these long-term changes that affect daily functioning are indicated as late effects in line with the definition of the Dutch Federation of Cancer Patient Organizations (Dutch Federation of Cancer Patient Organizations NFK 2017). Late effects of cancer treatment include, for instance, fatigue (Prue et al. 2006; Servaes et al. 2007; Reinertsen et al. 2010), lymphedema (Cormier et al. 2010), cardiovascular disease (Keating et al. 2006; Drafts et al. 2013), osteoporosis (Miller et al. 2016), anxiety (Mitchell et al. 2013), fear of recurrence (Lebel et al. 2016), or cognitive complaints (e.g. problems with concentration, learning and memory) (Wefel et al. 2015). Late effects of cancer treatment may continue to influence the ability to function at work for as long as ten or even more years after diagnosis (Koppelmans et al. 2012; Silver et al. 2013). The Dutch Federation for Cancer Patient Organizations reported that impairments resulting from these late effects were experienced in particular also in the context of work (Dutch Federation of Cancer Patient Organizations NFK 2017). This underlines the importance of studying late effects in the context of work.

To make comparisons possible it is necessary to study the associations of late effects of cancer treatment with a work outcome measure also used in studies among the general population or populations with chronic diseases. Therefore, a useful concept is ‘work ability’, which generally refers to the extent to which someone is able to carry out their work, taking the demands of the job, and health and mental resources into account (Ilmarinen et al. 2005). Work ability is reported to be a predictor of other work outcome measures among healthy populations, like absenteeism or early retirement (Ilmarinen and Tuomi 2004). In general, different (chronic) health problems are reported to be associated with decreased work ability (Leijten et al. 2014), and predictors of work ability are similar for workers with and without chronic health conditions (Koolhaas et al. 2013). However, other definitions are also used in the scientific literature (Lederer et al. 2014) and measurement methods of work ability may vary between studies (Brady et al. 2019; Cadiz et al. 2019). About a decade ago in an overview by Munir, Yarker, and McDermott (2009) on work ability and cancer, it was reported that very few well-validated measures of work ability had been used in previous studies. Therefore, it is important to report about the way work ability was assessed in the included studies within the current systematic literature review as well.

Furthermore, it is important to determine whether specific supporting factors in achieving work goals, so-called job resources within the Job Demands-Resources (JD-R) model (Demerouti et al. 2001), demonstrate an association with work ability in this specific population workers past cancer diagnosis or if job resources can even buffer a possible negative association of late effects of cancer treatment with a lower work ability. In the JD-R model, job demands are regarded as the aspects of the job that require effort and it is possible that the late effects of cancer treatment result in work demands being experienced as heavier. Furthermore, across studies among general populations job resources are positively related to work ability (Brady et al. 2019). In addition, in some studies job resources were reported to buffer the impact of job demands on burn-out (Bakker et al. 2005; Xanthopoulou et al. 2007). Clearly, job resources in the current work situation might be of great importance for work functioning among workers experiencing any late effects of cancer treatment after they returned to work.

As there is a shift in labor markets towards more flexible contracts, and smaller enterprises, the subpopulation of self-employed, freelancers and entrepreneurs, in other words the non-salaried, grows in several European Union member states (CBS 2019). These workers show different behavior after a cancer diagnosis than the salaried (Torp et al. 2018), as they more often continue working during treatment and take fewer time off work due to cancer. This might be due to the financial necessity to earn an income. Another difference is that the non-salaried have neither an employer, a supervisor, a human resource manager, an occupational physician, nor colleagues to provide job resources such as social support.

In short, this systematic literature review will focus on the work ability of all people working after a cancer diagnosis and cancer treatment (salaried and non-salaried). The aim is to present an overview of the studies that present data on work ability, also reporting on the method used to assess work ability. Furthermore, any available results on a possible association of late effects (physical complaints, fatigue or cognitive complaints) and work ability beyond the first 2 years after diagnosis will be reviewed. Finally, the role of job resources will also be evaluated.

## Methods

### Search strategy

To structure this systematic literature review the checklist of Preferred Reporting Items for Systematic Reviews and Meta-Analyses (PRISMA) was used (Moher et al. 2009). Systematic searches for publications were conducted on March 10th, 2020 in the databases PsycINFO, Medline, Business Source Premier and CINAHL, and on March 13th, 2020 in the databases ABI/Inform, Cochrane Library and Web of Science. Search terms were determined by the first author and an information specialist in mutual agreement with the other authors. In general, the search consisted of search terms for cancer combined with search terms for paid work. Search terms were broad to ensure no relevant studies would be missed. No restrictions were placed on publication date. For full search strategies, see Appendix 1. Additional searches consisted of citation tracking by the first author to discover articles not found by the systematic search.

*Inclusion criteria:* considered studies had to (1) be published in English peer-reviewed journals, (2) be an original quantitative research article (including pilot studies), (3) focus on work ability in people working after a cancer diagnosis, and (4) include adults (18 years or older).

*Exclusion criteria:* articles were excluded if they focused on (1) work-related risk factors for cancer, or (2) the ability to work if regarded as the ability to be at work rather than in the sense of work ability during work, or (3) populations entirely without paid work, or (4) populations entirely on long term sick leave, or (5) predicting return to work by work ability, or (6) the assessment of the effect of an intervention regarding return to work after a cancer diagnosis.

### Study selection

First, after the removal of duplicates, the search results were screened by title and abstract in Rayyan (Ouzzani et al. 2016) independently by the first author and two other researchers (the second author and research trainees). Those papers clearly not relevant to this review were eliminated. In case of a missing abstract or missing relevant details needed for screening, full paper copies were retrieved and screened. Second, the then included papers were used for additional citation tracking by the first author to identify possible additional studies. Third, the three authors discussed the eligibility of the remaining papers based on the criteria for inclusion and exclusion.

### Data extraction

After this, the first author extracted a range of data from the included papers relevant for this review, including data on (1) study design, (2) population (e.g. number of participants included in analyses, age, gender, cancer type, time since cancer diagnosis), (3) setting, (4) the assessment method of work ability, (5) possible late effects of cancer treatment, namely physical complaints, fatigue, and cognitive complaints, and (6) possible job resources (leadership style, coaching, organizational culture, social support, and autonomy). This data-extraction was reviewed by the second and the third author.

## Study characteristics

**Fig. 1:**
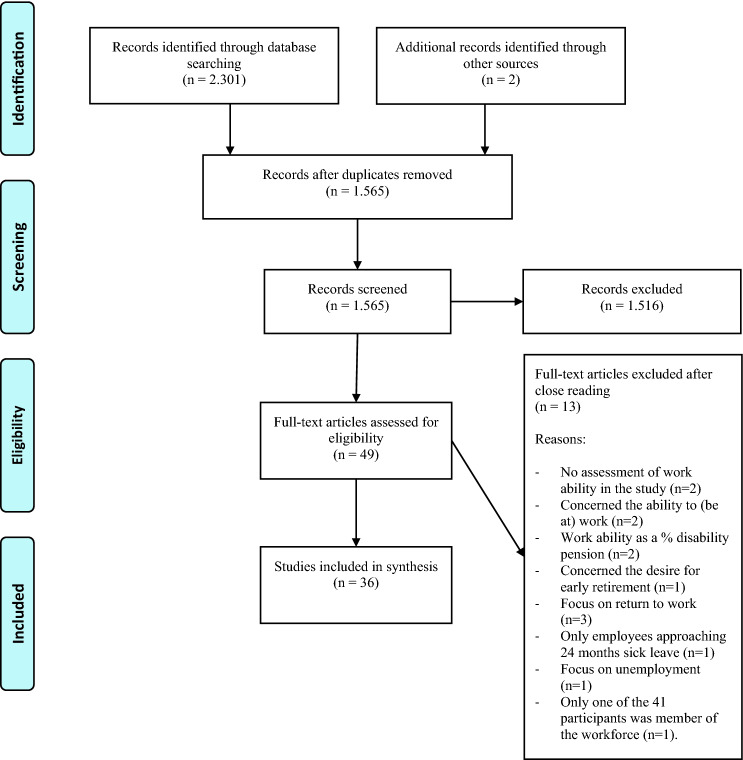
PRISMA 2009 flow diagram

The searches included 2303 records, including two results by additional citation tracking. After the removal of duplicates, 1565 titles and abstracts were screened. After elimination of the studies clearly not relevant to this review and after close reading 36 studies remained. A reason for this decrease in numbers was that studies on cancer and work mostly concern whether people return to work during the first 2 years after diagnosis and that these studies also focus on many other work-related aspects other than work ability. The study selection is documented in a PRISMA flow diagram, see Fig. 1. The data-extraction of the 36 studies is presented in Table 1.

**Table 1:** Summary of study results on the work ability in (self-)employed populations with a past cancer diagnosis

Authors and year of publication (reference number)	Study population	Study design	Work ability		Late effects of cancer treatment and work ability (> 2 years after diagnosis)			Job resources and work ability
	Type of cancer, *N* = (ex-) cancer patients in analysis, (gender), age in years, (% at work, type of employment), setting	Study approach and time points measured	Assessment method	Results in general	Physical complaints	Fatigue	Cognitive complaints	Job resources: social support, leadership style, coaching, autonomy, organizational culture
Bains et al. (2012)	Colorectal, primary diagnosis with curative treatment, *N* = 49 at T2, 44% female, mean age 52.49 (SD 5.42), 39% working at T2, United Kingdom	Longitudinal, T0 = post-surgery/pre-treatment, T1 = 3 months, T2 = 6 months	WAI item 1 is described (the method refers to three items)	Item 1: High work ability at baseline was associated with greater work ability at follow-up (*β* = 0.67,* t* = 3.99, *p* = .0005, f^2^ = 0.53)				
Bielik et al. (2020)	Ovarian, 13.8% metastatic, *N* = 123, female, mean age 59.7, 34.1% currently employed, Slovakia	Cross-sectional, mean 3.13 years after diagnosis	Current work ability 1 (worst)–10 (best) work ability covered by different dimensions surveys	Current work ability: Full health: 9.58 Without cancer: 9.07^*^ At diagnosis: 4.20^*^ At time of survey: 6.22 *Significant difference *p* < .001				
Carlsen et al. (2013)	Breast, *N* = 170, recurrence excluded, female, mean age 54.2 (range 42–64), controls *N* = 391, Denmark	Case–control, 5–8 years after diagnosis	WAI item 1	Item 1: mean 8.66 (controls 8.99), *p* < .0001		Fatigue (often), was associated with reduced work ability in a fully adjusted model (also controlled for health-related factors) (OR 10.7, CI 3.31–34.3) [stronger as among controls, 4.11 (1.97–8.57)]		Less help and support from a supervisor was significantly associated with reduced work ability (OR 2.40; CI 1.04–5.54) among the cancer survivors in the full model (also controlled for health-related factors). The latter was not the case for help and support from colleagues, but when only controlled for age this support showed a significant association (OR 3.47, CI 1.73–6.97)
Cheung et al. (2017)	Breast, primarily diagnosed, *N* = 151, mean age 49.98 (range 22–66), 43.1% currently working, 9.7% self-employed, Hong Kong	Cross-sectional, 1–16 years after diagnosis Work ability before diagnosis, during treatment and currently reported at time of survey	WAI items, 1, 2, 3, and 6	Item 1: work ability before diagnosis mean 8.48, SD 1.26, during treatment mean 4.95, SD 2.91, current mean 7.21, SD 1.81 Item 2 physical work ability (*N* = 54): 7.4% very good, 1.1% good, 64.8% moderate, 13.0% poor Item 2 mental work ability (*N* = 55): 10.9% very good, 45.5% good, 36.4 moderate, 5.5% poor 1.8 very poor Item 6 35% of the currently working not sure if they could continue to work in the subsequent 2 years Work ability before the diagnosis and work ability during treatment were associated with current work ability (0.63, *p* = .005 resp. .49, *p* < .0001) higher current work ability if less effects of health-related problems				Control at work was correlated with current work ability (Spearman’s rho 0.29, *p* = .038)
Couwenberg et al. (2020)	Rectal, *N* = 172, 8,7% metastatic, 71% male, median age 57, 100% paid employment, Dutch	Prospective cohort study (survey before treatment, 3, 6, 12, 18, and 24 months after treatment) controls *N* = 58	WAI	Significant decrease at 3, and 6 months Significantly lower than controls at 24 months				
Dahl et al. (2020)	Prostate, *N* = 730, 100% male, mean age 65.5 (*SD* 5.9), 46% working at time of survey, Norway	Cross-sectional, 3 years (*SD* 1.4) after treatment	WAI item 1	Current work ability 7.4 (*SD* 2.1)				
Dahl et al. (2016)	Prostate, *N* = 563, mean age 62.6 (*SD* 5.38) with 66% < 65 years, 93% working at time of survey, Norway	Cross-sectional, merge of national prospective study (questionnaires at baseline, 3, 12 and 24 months) and a cross-sectional single-hospital based survey, performed up to 6 years after radical prostatectomy	WAI items 1 and 2	Item 1 (*N* = 563): 8.6 (SD 0.5) Score 10: 30%, 8–9: 46%, 6–7: 15%, 0–5: 9% Item 2 (*N* = 542) physical work ability 55% very good, 28% pretty good, 13% fairly good, 3% quite bad, 1% very bad Item 2 (*N* = 539) mental work ability: 56% very good, 28% pretty good, 12% fairly good, 3% quite bad 1% very bad				
Dahl et al. (2019)	Breast, colorectal, leukemia, non-Hodgkin lymphoma, melanoma 63% female, median age 49 years (range 27–65), *N* = 1189, 75% employed (3% sick leave), Norway	Cross-sectional, median time since first cancer diagnosis was 16 years (range 6–31)	WAI item 1	Current work ability 8.3 (SD 1.8) among employed	Those with low work ability reported significantly higher mean levels of general health *p* < 0.001	Those with low work ability reported significantly higher mean levels of total fatigue* p* < 0.001		
De Boer et al. (2011)	Esophageal, stomach, colorectal, hepatic, pancreatic or biliary, new patients, 22% female, mean age 56 (SD 8), *N* = 333, 95 (self-) employed of whom 45 participated, the Netherlands	Cross-sectional, before treatment	WAI items 1 and 2	Item 1: mean current work ability was 5.4; for the subgroup not on sick leave higher (7.1, SD 2.7), than for the subgroup on sick leave (3.7, SD 2.2), *p* < .001 Item 2: Physical work ability and mental work ability higher for the group not on sick leave				
De Boer et al. (2008)	Breast, female genitals or genito-urological mostly, primary diagnosis of cancer, *N* = 195 at T3 (24% already returned to work at 6 months), 60% female, mean age 42.2 (*SD* 9.3), the Netherlands	Longitudinal (prospective), T1 = 6 months after first day of sick leave, T2 = 12 months after first day of sick leave, T3 = 18 months after first day of sick leave	WAI items 1 and 2	Item 1: significant rise in scores from T1 to T2 and from T2 to T3 (4.6, *SD* 3.2, 6.3, *SD* 2.7, and 6.7, *SD* 2.7 resp.) Both men and women improved over time (*p* < .001), but women improved more (*p* = .002) Patients with cancer of the female genitals and breast cancer patients improved most over time (*p* = .01)				
Doll et al. (2016)	Uterine, ovarian, cervical, vulvar, and other (only new), and also benign disease, *N* = 185 at baseline, female, mean age 56.5 (SD 13), *N* = 174 at T3, United States of America	Longitudinal (prospective), T1 = 1 month after surgery, T2 = 3 months after surgery, T3 = 6 months after surgery	A subset of questions of the WAI, in this study item 1 is used	Item 1: Baseline without surgical complications 8.8 (*SD* 2.3), with surgical complications 7.7 (*SD* 3.2)				
Duijts et al. (2017)	Various (48% breast), part 1 of the study: *N* = 252, 69.8% female, mean age 50.7 (SD 7.4) at T0, all with employment contract) at T2, self-employed, temporary agency workers and workers without an employment contract excluded, The Netherlands	Longitudinal (prospective), T0 = 2 years after diagnosis, T1 = 3 years after diagnosis, T2 = 4 years after diagnosis	WAI item 1	Item 1: Group N = 151 ‘continuously working’ 5.6 (SD 1.8) Multivariate time lag model: current work ability predictor of work continuation one year later (*p* = .007), ß = 0.38 (SE 0.14)/ OR 1.46; CI 1.11–1.92)				
Fosså et al. (2015)	Prostate, *N* = 612 (30% working), mean age 69 (range 47–105, with 30% < 65) Norway	Cross-sectional, median observation time since diagnosis 4.0 years (range, 0–23 years)	Self-reported reduction of work ability (“no”: score of 0–5 vs. “yes”: score of 6–10)	Limitations of work ability: 10–22%	Significantly fewer patients experienced limitations of their work ability after radical prostatectomy (10%) than after high-dose radiotherapy (22%)			
Gregorowitsch et al. (2019)	Breast, *N* = 939 (68% employed at baseline, median age 52), The Netherlands	Prospective cohort study (baseline, 6, 18, and 30 months) Controls *N* = 3,641	WAI	Employed: baseline 71% moderate-poor work ability 30 months 24% moderate-poor work ability (lower than controls)				
Gudbergsson et al. (2008a)	Breast, testicular, or prostate, *N* = 446 (all returned to work), 51% female, age 49.1 (SD 9.3), (also self-employed) and norm group *N* = 588, Norway	Case–control 2–6 years after primary surgery or chemotherapy	WAI items 1, 2 and 3	Item 1: Survivors scored lower (mean 8.2, SD 2.0) than norm group (mean 8.6, SD 1.6), *p* < .001, effect size 0.25 Item 2: Survivors scored more moderate/rather poor/poor physical work ability (21% versus 9%, *p* < .001, effect size 0.34) and more moderate/rather poor/poor mental work ability (19% versus 9%, *p* < .001, effect size 0.30)				Survivors experienced more support from colleagues at work (*p* = .005), but similar control as the norm group No data on possible associations of these factors with work ability reported
Gudbergsson et al. (2008b)	Breast, testicular, or prostate, first cancer diagnosis between 25–57 years of age, *N* = 513, 51% female, 84% had returned to work, and of this group 83% had no work changes and 17% did have work changes, Norway	Cross-sectional, 2–6 years after primary treatment	WAI items 1, 2, and 3	Item 1: the subgroup with work changes scored lower (mean 6.9, SD 2.4) than group without work changes (mean 8.5, SD 1.8), p < .001, effect size 0.75 Item 2: The subgroup without work changes scored less low (moderate, rather poor, poor) on physical work ability (16% versus 38%) and mental work ability (14% versus 30%) than the subgroup with work changes (both *p* < .001, effect sizes 0.51 and 0.61) Mental work ability (and not physical work ability) reduced due to cancer was associated with current work ability in univariate and multivariate analyses (ß −0.139, *p* = .003)	Symptom scale score was associated with current work ability in univariate analyses (ß = 0.396, *p* < .001)			Social support from colleagues was associated with current work ability in univariate analyses (ß = 0.241, *p* < .001) No data on possible association of control with work ability reported
Gudbergsson et al. (2011)	Breast, testicular, or prostate, *N* = 446, 52% female, mean age 52.9 (SD 6.5), and control group *N* = 588, Norway	Case control, 2–6 years after primary treatment	WAI items 1, 2 and 3	Item 1: males had a higher work ability (8.4, SD 1.8) than females (8.0, SD 2.1), *p* = .04, effect size = 0.20) No gender differences in control group (8.6, SD 1.6) Item 2: No difference in physical work ability or mental work ability between male and female survivors Difference between male survivors and male controls on physical work ability (effect size 0.37, *p* < .001) and mental work ability (effect size 0.27, *p* = .004) Difference between female survivors and female controls on mental work ability (effect size 0.30, *p* < .001). No gender difference between female survivors and female controls on physical work ability	Somatic symptoms were associated with overall current work ability in univariate analyses and multivariate analyses (ß =−0.078, *p* = .012)			Support from colleagues and supervisors was assessed and combined with communication No separate data of an association of only social support with overall current work ability
Hartung et al. (2018)	Hematological, *N* = 91 at baseline, 67% male, mean age 49 (*SD* 8), *N* = 52 at T1, *N* = 40 at T2, 10% self-employed, Germany	Longitudinal, baseline (less than 4 weeks before treatment), 6 months, and 1 year	WAI	Mean WAI significantly increased from 18.5 at baseline to 28.3 after 12 months (*p* = 0.001)				
Ho et al. (2018)	Breast, *N* = 327, female, 6% recurrent disease, mean age at time of diagnosis: 47 (range 42–52), mean age at time of survey: 53 (range 48–58), 53% employed, Singapore	Cross-sectional, 3–8 years after diagnosis	WAI	Item 1 *N* = 168 employed: work ability 8% poor, 29% moderate, 48% good, and 15% excellent	Survivors with suboptimal work ability expressed more breast and arm symptoms, as compared with survivors with good or excellent work ability	General, physical, and mental fatigue were less common in survivors with optimal work ability Higher level of physical fatigue remained significant-ly associated with poorer work ability in the full model	Breast cancer survivors with suboptimal current workability had lower scores for cognitive functioning	
Kiserud et al. (2016)	Lymphoma. *N* = 312, also second cancers, 85% working or on sick leave at baseline and 58% at moment of survey, 40% female, mean age 41.5 (SD 13.5) at diagnosis and 54.0 (SD 11.3) at time of survey, Norway	Cross-sectional follow-up study, mean time from diagnosis to survey was 12.4 years (SD 6.1) and from HDT-ASCT to survey 9.7 years (SD 5.1)	WAI items 1 and 2	Item 1: The subgroup employed at follow up: 9.2 (SD 1.8) at diagnosis and 7.3 (SD 2.5) at moment of survey				
Lee et al. (2008)	Stomach, *N* = 408, 73.5% male, also self-employed and not-working included, also 994 general population, Korea	Case control, 21–36 months after diagnosis	Multiple-choice item regarding lessened work-related ability than before cancer diagnosis	More cancer survivors had lessened work-related ability (37%) than the general population (10.6%), OR 6.11, CI 3.64–10.27		Easily fatigued and exhausted in the workplace: 50% of the cancer survivors versus 22.4% in the general population (OR 4.02, CI 2.55–6.33) No data on the association with work ability		
Lindbohm et al. (2012)	Breast, testicular, prostate, or lymphoma, *N* = 1449, 66% female, age 25–57 at time of diagnosis, reference group *N* = 2709, Denmark, Finland, Iceland, and Norway (in the Iceland sample cancer recurrence excluded)	Case control, 1–8 years after diagnosis	WAI item 1	Item 1: age-adjusted mean work ability was slightly lower among the breast cancer survivors (8.41) than among the female reference group (8.58, *p* < .01). No difference in work ability between men with testicular cancer diagnosis (8.76) and the male reference group (8.69). Prostate cancer survivors had a lower work ability (8.28) than the male reference group (*p* < .01)				Low support from supervisor or colleagues were associated with low work ability among both men and women, in the cancer group and the reference group High colleagues’ avoidance behavior was related to lower work ability among female cancer survivors (*p* < .001) (and not in female references) Supervisors’ high avoidance behavior was related to lower work ability among male cancer survivors (*p* < .01) (and not in references) No data of an association of social climate with work ability
Moskowitz et al. (2014)	Breast, testicular, colorectal, and prostate cancer, Hodgkin lymphoma and non-Hodgkin Lymphoma, among others, *N* = 1525, 15.8% recurrence or secondary cancer, 61.6% female, mean age 49.1 (SD 10.8), also self-employed included, United States of America	Cross-sectional, average time since completion of treatment was 3 years (range 0–464 months)	Whether unable to work full time, unable to work the same as before cancer, or unable to work at all		A greater level of functional limitations (physical, cognitive and social) were significantly related to limited work ability (*β* = 5.88, *p* < .001)	A greater level of functional limitations (physical, cognitive and social) were significantly related to limited work ability (*β* = 5.88, *p* < .001) A greater level of symptoms (cognitive, distress, fatigue, cancer fear, family fear) were not significantly related to limited work ability	A greater level of symptoms (cognitive, distress, fatigue, cancer fear, family fear) were not significantly related to limited work ability	
Musti et al. (2018)	Breast, *N* = 503, mean age 51.5 (SD 3.6), permanent, fixed term and other type of contract, Italy	Cross-sectional, survey 3.2 (SD 0.9) years since treatment, retrospective about moment return to work (23.0% experienced > 6 months sick leave)	Same or reduced work ability	43.5% reduced work ability at moment of return to work				Support/solidarity from employer 85.1% in group with no reduced work ability and 70.2% in group with reduced work ability, *p* < 0.001 Support/solidarity from colleagues 91.5% in group with no reduced work ability and 76.8% in group with reduced work ability, *p* < 0.001
Neudeck et al. (2017)	Thyroid, *N* = 66, 69.7% female, 68% working, Switzerland	Cross-sectional, max. 7 years after treatment. Mean time since the diagnosis of thyroid cancer was 37.8 months (SD: 21.7; range: 7–79)	Ad hoc question-naire	71.2% felt impaired with respect to their work ability during the first year after the diagnosis				
Nieuwenhuijsen et al. (2009)	Gastrointestinal, breast, female genitals, male genitals, urological haematological, and other types, primary diagnosis of cancer, *N* = 195 at T1 (of whom *N* = 45 neuropsychological tested at T2), 67% female, mean age 44 (SD 9), the Netherlands	Longitudinal (prospective), T1 = 6 months after first day of sick leave, T2 = 12 months after first day of sick leave, also neuro-psychological testing, T3 = 18 months after first day of sick leave	WAI item 1 on T2	Item 1: At T1 no difference (*p* = .27) between the participants in the neuro- psychological study (4.1, SD 3.0) and the rest of the cohort (4.7, SD 3.3)				
Nilsson et al. (2016)	Breast, female, *N* = 692 at T1, mean age 50.8 (SD 8.07), Sweden	Longitudinal (prospective), T1 = 4 weeks after surgery T2–T6 during 24 months	WAI item 2	Item 2: significant difference in physical work ability between baseline (*β* = 0.354, *p* < .001) and 4 months (β = 0.138, *p* < .001) as well as between 4 and 8 months (*β* = 0.285, *p* < .001) Item 2: significant differences in mental/social work ability were found between 8 and 12 months (*β* = 0.286, *p* < .001)				
Ortega et al. (2018)	Breast, *N* = 114 (three treatment groups of *N* = 38), female, mean ages 48.1–50.1, self-employed 36.8–52.6%, Brazil	Cross-sectional, > 1 year after treatment	Work Limitations Questionnaire (the percentage of time limited in performing work tasks in the last 2 weeks)	Patients in the mastectomy and breast-conserving surgery groups showed reduced work effectiveness (presenteeism) and loss of productivity compared with women in the breast reconstruction and control groups (*p* = 0.0004 and *p* = 0.0006, respectively)				
Tamminga et al. (2019)	Breast (61%), gynecological cancer (35%), or other type of cancer (4%) Intervention group *N* = 49, mean age 47.1 (SD 8.2), 98% female Control group *N* = 57, mean age 47.8 (SD 7.6), 100% female, 4% self-employed, The Netherlands	Longitudinal, baseline and at 6, 12, 18, and 24 months of follow-up	WAI items 1 and 2	Work ability improved from baseline to 1 year and stable from 1 to 2 years				
Taskila et al. (2007)	Breast, lymphoma, testicular or prostate, no distant metastasis, *N* = 591, 73,9% female, age 25–57 at time of diagnosis, also freelancers and entrepreneurs included, also 757 referents, Finland	Case control, 2–6 years after diagnosis	WAI items 1 and 2	Item 1: nearly the same as in referents and highest mean value for men with testicular cancer (8.95), and lowest for men with prostate cancer (8.00) Item 2: 26% reported deteriorated physical work ability due to cancer 19% reported deteriorated mental work ability due to cancer				Among the female survivors (and male referents, but not among male survivors), co-workers’ support was related to reduced risk of impaired physical work ability (OR 0.83, CI 0.73–0.94) and for impaired mental work ability (OR 0.84, CI 0.73–0.96) A better social climate at work was only related to impaired mental work ability (and not to physical work ability), for male survivors (OR 0.80, CI 0.70–0.91) and for female survivors (OR 0.84, CI 0.76–0.94)
Torp et al. (2012)	15 most common cancers: like breast, gynecological, prostate, testicular, *N* = 653, primary diagnoses, 9% with metastasis, 68% female, mean age 51.9 (SD 7.9), 6% self-employed, Norway	Cross-sectional, 15–39 months after cancer diagnosis	WAI items 1 and 2	Item 1: mean total (current) work ability was 8.6 (SD 1.8) among men and 8.6 (SD 1.7) among women Self-employment was a predictor for lower work ability. Comorbidity (36%) was strongly correlated with work ability Item 2: 31% reported a reduction in physical work ability due to cancer 23% reported a reduction in mental work ability. More women than men had reduced mental work ability due to cancer				General social support (*β* = 0.15, *p* ≤ .001) is a significant predictor of total work ability in univariate (and not in multivariate) regression Cancer-related colleague support was a significant predictor of total work ability (*β* = 0.15, *p* ≤ .01) in multivariate regression Cancer-related supervisor support was not a significant predictor of total work ability in regression analyses Decision latitude (*β* = 0.08, *p* ≤ .05) is a significant predictor of work ability in univariate (and not in multivariate) regression
Torp et al. (2017)	Most common invasive types of cancer: colon, rectal, lung, skin (melanoma), breast, cervical, uterine, ovarian, prostate, testicular, bladder, central nervous system, thyroid, non-Hodgkin lymphoma, and leukemia, *N* = 1115, 69% female, 8% self-employed Not returned to work at time of survey: 24% self-employed and 18% salaried	Cross-sectional, 15–39 months after diagnosis	WAI items 1 and 2	Item1: compared with the salaried workers, the self-employed people reported significantly more often reduced total work ability (*p* = .02, effect size 0.26). The negative effect of self-employment on total work ability seems to be mediated by reduced work hours and a negative cancer-related financial change Item 2: no significant differences between the salaried and the self-employed	Poor-self rated health status correlated significantly with low total work ability in logistic regression analyses			Having higher decision latitude at work was a factor preventing low total work ability (OR 0.80, CI 0.68–0.94)
Von Ah et al. (2018)	Breast, *N* = 68, exclusion of secondary cancers or metastasis, mean age 52.12 (SD 8.16), United States of America	Cross-sectional, study population on average 5 (SD 3.8) years post-treatment (minimum 1 year)	WAI	Mean 38.9 (SD 7.5). Poor or moderate work ability: 26.5%			Significant relationship between perceived cognitive impairment and work ability (*β* = − 0.658, *p* < .000) Explained variance: 46,5% Significant relationship between perceived cognitive ability and work ability (*β* = 0.472, *p* < .000) Explained variance: 29,9%	
Von Ah et al. (2017)	Breast *N* = 68, exclusion of brain metastasis, mean age 52.12 (*SD* 8.603), 1% self-employed, United States of America	Cross-sectional, study population on average 4.97 (SD 3.36) years post-treatment (minimum 1 year)	WAI	WAI: Mean 38.91 (*SD* 7.45) Poor 7–27: 10%, moderate 28–36: 16%, good 37–43: 46%, excellent 44–49: 28%			Linear regression: significant relationship between attentional fatigue (higher = higher level of attention) and perceived work ability (ß = 0.627, *p* < .001), Explained variance: 39%	
Wolvers et al. (2019)	Breast 84%, colorectal, Non-Hodgkin, lymphoma, other, *N* = 89, 91% female, mean age 47.9 (7.2), 10% self-employed, The Netherlands	Longitudinal intervention study, baseline, 6, 12, 18 months	WAI item 1	Inverse, longitudinal association between fatigue and perceived work ability				
Zanville et al. (2016)	Breast, *N* = 44 (22 chemo-therapy-treated and 22 chemo-therapy-naïve), non-metastatic, female, mean age resp. 49.68 (SD 8.0) and 52.68 (SD 9.3), United States of America	Longitudinal, T0 = pre-treatment (approximately one third of chemotherapy-treated received neo-adjuvant chemotherapy and were surgery and treatment naïve at baseline), T1 = approximately 1-month post-chemotherapy, T2 = approximately 1 year after T1	Item from Functional Well-Being subscale of FACT/GOG-Ntx (version 4)	–				

*N *Number, *SD* Standard Deviation, *OR *Odds Ratio, *CI *Confidence Interval

The 36 studies covered 12 (33%) longitudinal studies (De Boer et al. 2008; Nieuwenhuijsen et al. 2009; Bains et al. 2012; Nilsson et al. 2016; Doll et al. 2016; Zanville et al. 2016; Duijts et al. 2017; Hartung et al. 2018; Wolvers et al. 2019; Gregorowitsch et al. 2019; Tamminga et al. 2019; Couwenberg et al. 2020), six (17%) case–control studies (Taskila et al. 2007; Gudbergsson et al. 2008a, 2011; Lee et al. 2008; Lindbohm et al. 2012; Carlsen et al. 2013), and 18 (50%) cross-sectional studies. Almost half of all included studies was published in 2017 or later. The setting of 14 studies was Northern Europe. Other European settings were the Netherlands (eight studies), and the United Kingdom, Germany, Italy, Switzerland, and Slovakia with one study each. Other settings outside Europe were the United States of America (five studies), Brazil (one study), and Asia (three studies). The studies focused on a combination of types of cancer in 16 studies, breast cancer in ten studies, prostate cancer in three studies, and ovarian, rectal, colorectal, thyroid, stomach cancer, hematological cancer and lymphoma in one study each. Gender was not mentioned in five studies (14%) among populations with a past breast cancer diagnosis, very likely to be women but possibly not all, and not in two studies among prostate cancer diagnoses, the latter certainly concerning men. The gender distribution therefore showed eight studies (22%) among women, five (14%) not with full certainty only among women, three studies (8%) among men, and 20 studies (56%) among both genders. Type of employment was not clear in 16 studies (44%). The other 20 studies concerned 13 studies (36%) with both employed and self-employed, 7 studies with employed only (20%), and none of the studies only included self-employed. The baseline of the data collection varied from the moment of diagnosis, the first day of sick leave, to the end of primary treatments.

## Quality assessment

The methodological quality of the studies was assessed using three quality assessment checklists. For cohort and case–control studies the checklists from the ‘Critical Appraisal Skills Program’ (CASP) were used (Critical Appraisal Skills Program 2018a, b). Some items were adapted to the current study. These adjustments are described in the notes below the Tables 2, 3, and 4. For cross-sectional studies (except case–control studies) the Appraisal tool for Cross Sectional Studies (AXIS tool) (Downes et al.) was used. The quality assessment was used to test the quality across studies.

**Table 2:** Quality assessment for the cohort studies by means of the checklist from the ‘Critical Appraisal Skills Program’ (CASP)

	Bains et al. (2012)	De Boer et al. (2008)	Couwenberg et al. (2020)	Doll et al. (2016)	Duijts et al. (2017)	Gregorowitsch et al. (2019)	Hartung et al. (2018)	Nieuwenhuijsen et al. (2009)	Nilsson et al. (2016)	Tamminga et al. (2019)	Wolvers et al. (2019)	Zanville et al. (2016)
1. Did the study address a clearly focused issue?	Yes	Yes	Yes	Yes	Yes	Yes	Yes	Yes	Yes	Yes	Yes	Yes
2. Was the cohort recruited in an acceptable way?	Yes	Yes	Yes	Yes	Yes	Yes	Yes	Yes	Yes	Yes	Yes	Yes
3. Was the exposure accurately measured to minimize bias?	Yes	Yes	Yes	No Benign tumors included	Yes	Yes	Yes	Yes	Yes	Yes	Yes	Yes
4. Was work ability accurately measured to minimize bias?	Yes	Yes	Yes	Yes	Yes	Yes	Yes	Yes	Yes	Yes	Yes	Yes
5. Have the authors identified all important confounding factors?	Yes	N.a	Yes	N.a	N.a	N.a	Yes	N.a	N.a	Yes	Yes	Yes
6. Have they taken account of the confounding factors in the design and/or analysis?	Yes	N.a	Yes	N.a	N.a	N.a	Yes	N.a	N.a	Yes	Yes	Yes
7. Was the follow up of subjects complete enough?	Yes	Yes	Yes	N.a.	N.a	Yes	Yes	N.a	N.a	Yes	Yes	Yes
8. Was the follow up of subjects long enough to investigate late effects?	No (6 months)	No (18 months)	No (24 months)	No (6 months)	Yes	Yes	No (12 months)	No (18 months) Sub study was cross-sectional	No (2 years)	No (2 years)	No (18 months)	No (1 year)
9. What are the results of this study?	See Table 1	See Table 1	See Table 1	See Table 1	See Table 1	See Table 1	See Table 1	See Table 1	See Table 1	See Table 1	See Table 1	See Table 1
10. Are the results precise?	Yes	Yes	Yes	Yes	Yes	Yes	Yes	Yes	Yes	Yes	Yes	Yes
11 Do you believe the results?	Yes	Yes	Yes	Yes	Yes	Yes	Yes	Yes	Yes	Yes	Yes	Yes
12. Can the results be applied to the local (European) population?	Yes	Yes	Yes	No (USA)	Yes	Yes	Yes	Yes	Yes	Yes	Yes	No (USA)
13. Do the results of this study fit with other available evidence with regard to work ability?	Yes	Yes	Yes	N.a	N.a	Yes	Yes	N.a	N.a	Yes	Yes	Yes
14. What are the implications of this study for practice?	See Table 1	See Table 1	See Table 1	See Table 1	See Table 1	See Table 1	See Table 1	See Table 1	See Table 1	See Table 1	See Table 1	See Table 1

(a) Because of the aim of the systematic literature review in questions 3 a past cancer diagnosis was regarded as exposure

(b) Because of the aim of the systematic literature review questions 5 and 6 were answered in the case that ‘work ability’ was an outcome measure. In all other cases ‘N.a.’ was noted

(c) Because of the aim of the systematic literature review in question 8 the criterion is two years

(d) In question 7 and 13 the quality assessment is only made in the case of measurements of the level of work ability at different time points. In all other cases ‘N.a.’ was noted

(e) In question 8 ‘late effects?’ was added

(f) Question 10 was rephrased

(g) In question 12 Europe was regarded as the region of the local population

(h) In question 13 ‘with regard to work ability’ is added

**Table 3:** Quality assessment for the case–control studies by means of the checklist from the ‘Critical Appraisal Skills Program’ (CASP)

	Carlsen et al. (2013)	Gudbergsson et al. (2008a)	Gudbergsson et al. (2011)	Lee et al. (2008)	Lindbohm et al. (2012)	Taskila et al. (2007)
1. Did the study address a clearly focused issue?	Yes	Yes	Yes	Yes	Yes	Yes
2. Did the authors us an appropriate method to answer their question?	Yes	Yes	Yes	Yes	Yes	Yes
3. Were the cases recruited in an acceptable way?	Yes	Yes	Yes	Yes	Yes	Yes
4. Were the controls selected in an acceptable way?	Yes	Yes	Yes	Yes	Yes	Yes
5. Was the exposure accurately measured to minimize bias?	Yes	Yes	Yes	Yes	Yes	Yes
6. Aside from the experimental intervention (cancer–no cancer), were the groups treated equally?	Yes	Yes	Yes	Yes	Yes	Yes
7. Have the authors taken account of the potential confounding factors in the design and/or in their analysis?	Yes	Yes	Yes	Yes	Yes	Yes
8. How large was the treatment (cancer–no cancer) effect?	See Table 1	See Table 1	See Table 1	See Table 1	See Table 1	See Table 1
9. How precise was the estimate of the treatment effect?	N.a	N.a	N.a	N.a	N.a	N.a
10. Do you believe the results?	Yes	Yes	Yes	Yes	Yes	Yes
11. Can the results be applied to the local (European) population?	Yes	Yes	Yes	No (Korea)	Yes	Yes
12. Do the results of this study fit with other available evidence?	Yes	Yes	Yes	Yes	Yes	Yes

(a) Because of the aim of the systematic literature review in questions 6 and 8 ‘(cancer–no cancer)’ was added

(b) Question 9 was not applicable as there is no treatment effect involved

(c) In question 11 Europe was regarded as the region of the local population

**Table 4:** Quality assessment for the cross-sectional studies by means of the Appraisal tool for Cross Sectional Studies (AXIS tool)

	Bielik et al. (2020)	Cheung et al. (2017*)*	Dahl et al. (2020)	Dahl et al. (2016)	Dahl et al. (2019)	De Boer et al. (2011)	Fosså et al. (2015)	Gudbergsson et al. (2008b)	Ho et al. (2018)	Kiserud et al. (2016)	Moskowitz et al. (2014)	Musti et al. (2018)	Neudeck et al. (2017)	Ortega et al. (2018)	Torp et al. (2012)	Torp, Syse et al. (2017)	Von Ah et al. (2018)	Von Ah et al. (2017)
1. Were the aims/objectives of the study clear?	Yes	Yes	Yes	Yes	Yes	Yes	Yes	Yes	Yes	Yes	Yes	Yes	Yes	Yes	Yes	Yes	Yes	Yes
2. Was the study design appropriate for the stated aim(s)?	Yes	Yes	Yes	Yes	Yes	Yes	Yes	Yes	Yes	Yes	Yes	Yes	Yes	Yes	Yes	Yes	Yes	Yes
3. Was the sample size justified?	Yes	Yes	Yes	Yes	Yes	Yes	Yes	Yes	Yes	Yes	Yes	Yes	Yes	Yes	Yes	Yes	Yes	Yes
4. Was the target/reference population clearly defined? Is it clear who the research was about?	Yes	Yes	Yes	Yes	Yes	Yes	Yes	Yes	Yes	Yes	Yes	Yes	Yes	Yes	Yes	Yes	Yes	Yes
5. Was the sample frame taken from an appropriate population base so that it closely represented the target/reference population under investigation?	Yes	Don’t know. Convenience sample from three sources	Yes	Yes	Yes	Yes	Yes	Yes	Yes	Yes	Yes	Yes	Yes	Yes	Yes	Yes	Yes	Yes
6. Was the selection process likely to select subjects/participants that were representative of the target/reference population under investigation?	Don’t know. Partly pilot study	Yes	Yes	Yes	Yes	Yes	Yes	Yes	Yes	Yes	Yes	Yes	Don’t know. Pilot study	Yes	Yes	Yes	Yes	Yes
7. Were measures undertaken to address and categorize non-responders?	Don’t know. No information	Yes	Yes	Don’t know Data from other studies	N.a	Yes	No	Yes	Yes	Yes	No	Yes	No	Don’t know. No information	No	No	No	No
8. Was work ability measured appropriate to the aims of the study?	Mixed: current work ability appropriate, work ability unclear	Yes	Yes	Yes	Yes	Yes	Yes	Yes	Yes	Yes	Yes	Yes	Yes	Yes	Yes	Yes	Yes	Yes
9. Was work ability measured correctly using instruments/measurements that had been trailed, piloted or published previously?	Don’t know	Yes	Yes	Yes	Yes	Yes	Don’t know. No information	Yes	Yes	Yes	Don’t know. No information	Don’t know. No information	No A non-validated ad hoc questionnaire	Yes	Yes	Yes	Yes	Yes
10. Is it clear what was used to determined statistical significance and/or precision estimates? (e.g., p values, CIs)	Yes	Yes	Yes	Yes	Yes	Yes	Yes	Yes	Yes	Yes	Yes	Yes	Yes	Yes	Yes	Yes	Yes	Yes
11. Were the methods (including statistical methods) sufficiently described to enable them to be repeated?	Yes	Yes	Yes	Yes	Yes	Yes	Yes	Yes	Yes	Yes	Yes	Yes	Yes	Yes	Yes	Yes	Yes	Yes
12. Were the basic data adequately described?	Yes	Yes	Yes	Yes	Yes	Yes	Yes	Yes	Yes	Yes	Yes	Yes	Yes	Time since diagnosis unclear	Yes	Yes	Yes	Yes
13. Does the response rate raise concerns about non-response bias?	No	No	No	No	No	No	No	No	No	No	No	No	No	No	No	No	No	No
14. If appropriate, was information about non-responders described?	Yes	Yes	Yes	No	N.a	Yes	No	Yes	Yes	Yes	No	Yes	No	Yes	No	No	No	No
15. Were the results internally consistent?	Yes	Yes	Yes	Yes	Yes	Yes	Yes	Yes	Yes	Yes	Yes	Yes	Yes	Yes	Yes	Yes	Yes	Yes
16. Were the results for the analyses described in the methods, presented?	Yes	Yes	Yes	Yes	Yes	Yes	Yes	Yes	Yes	Yes	Yes	Yes	Yes	Yes	Yes	Yes	Yes	Yes
17. Were the authors’ discussions and conclusions justified by the results?	Yes	Yes	Yes	Yes	Yes	Yes	Yes	Yes	Yes	Yes	Yes	Yes	Yes	Yes	Yes	Yes	Yes	Yes
18. Were the limitations of the study discussed?	Yes	Yes	Yes	Yes	Yes	Yes	Yes	Yes	Yes	Yes	Yes	Yes	Yes	Yes	Yes	Yes	Yes	Yes
19. Were there any funding sources or conflicts of interest that may affect the authors’ interpretation of the results?	No	No	No	No	No	No	No	No	No	No	No	No	Don’t know. Pilot study	No	No	No	No	No
20. Was ethical approval or consent of participants attained?	No	Yes	Yes	Yes	Yes	Yes	Yes	Yes	Yes	Yes	Yes	Yes	Yes	Yes	Yes	Yes	Yes	Yes

(a) Because of the aim of the systematic literature review in questions 8 and 9 ‘work ability’ was inserted

The quality assessment was performed for all 36 studies by the first author. The second and the third author independently assessed the quality of different subsets of cohort, case–control and cross-sectional studies. The results were discussed afterwards, and agreement was reached on the level of quality of each of the included studies for the present study.

The 12 cohort studies were all of good quality and therefore no studies were excluded. Of the 12 included cohort studies two studies made use of a follow up period long enough to possibly investigate late effects of cancer treatment that is beyond two years after diagnosis (Duijts et al. 2017; Gregorowitsch et al. 2019). Furthermore, these two studies concerned European populations.

Also the six case–control studies were all of good quality, not resulting in any exclusions. The time since diagnosis was beyond 2 year after diagnosis in four studies and two studies also included participants within the first two years after diagnosis. Five studies of the case–control studies concerned European populations (Taskila et al. 2007; Berg Gudbergsson et al. 2008a, b; Gudbergsson et al. 2011; Lindbohm et al. 2012; Carlsen et al. 2013).

The 18 cross-sectional studies showed some quality differences, but the quality of all studies was acceptable. The selection process in two pilot studies might have impaired representativeness (Neudeck et al. 2017; Bielik et al. 2020). In one cross-sectional study the time since diagnosis was not clear (Ortega et al. 2018), but the other 17 cross-sectional studies concerned populations with participants beyond 2 years after diagnosis. For the checklists see Tables 2, 3 and 4.

## Assessment methods used to measure work ability

Six (17%) of the included studies (Von Ah et al. 2017, 2018; Ho et al. 2018; Hartung et al. 2018; Gregorowitsch et al. 2019; Couwenberg et al. 2020) used the complete Work Ability Index (WAI), a questionnaire that consists of seven items. These 7 items are (1) current work ability compared with the lifetime-best (on a scale of 0–10), (2) work ability in relation to the (physical and mental) demands of the job, (3) number of current diseases diagnosed by a physician, (4) estimated work impairment due to diseases, (5) sick leave during the past 12 months, (6) own prognosis of work ability two years from now, and (7) mental resources. Only partial use of the WAI (one or more items) was made by 22 (61%) studies, with the first WAI item being used in 21 studies (see Table 1).

Of the eight (22%) studies not using the complete or partial WAI, different ways to assess work ability were used, namely (1) the Functional Well-Being subscale of the FACT/GOG-Ntx (version 4) (Zanville et al. 2016), (2) a multiple-choice question regarding lessened work-related ability (Lee et al. 2008), (3) a self-reported reduction of work ability (Fosså and Dahl 2015; Musti et al. 2018), (4) a multiple choice question regarding being unable to work full time, unable to work the same as before cancer or unable to work at all (Moskowitz et al. 2014), (5) the Work Limitations Questionnaire (the percentage of time limited in performing work tasks in the last two weeks) (Ortega et al. 2018), (6) a question on current work ability in combination with other information (Bielik et al. 2020), and (7) a non-validated ad hoc questionnaire (Neudeck et al. 2017). In brief, 22% of the studies did not use the complete or partial WAI but other ways to assess work ability.

## Results: work ability in working people with a past cancer diagnosis

After a cancer diagnosis the level of work ability tended to be experienced as lower than before diagnosis. However, cohort studies demonstrated that the level of work ability among workers during the first two years past cancer diagnosis appeared to improve significantly (De Boer et al. 2008; Nilsson et al. 2016). One longitudinal study with a two year follow up reported work ability improved over time most prominently from baseline to 1 year of follow-up and thereafter remained stable up to 2 years of follow-up (Tamminga et al. 2019). However, other longitudinal studies that focused on the first two years did not have data on the course of work ability (Nieuwenhuijsen et al. 2009; Bains et al. 2012; Doll et al. 2016; Zanville et al. 2016), nor had the study with a follow-period of four years past cancer diagnosis (Duijts et al. 2017). However, compared to controls work ability was reported to be significantly lower when 2 years after diagnosis (Couwenberg et al. 2020).

Cross-sectional studies that used data reported by the respondents retrospectively with regard to different time points after cancer diagnosis, also reported that work ability was lowered after cancer diagnosis and experienced as increasing again (Kiserud et al. 2016; Cheung et al. 2017; Musti et al. 2018; Bielik et al. 2020). Some studies only focused on the association of different types of treatment and work ability (Ortega et al. 2018; Dahl et al. 2020). Furthermore, when the complete Work Ability Index (WAI) was used to assess work ability the results were as follows. Suboptimal work ability was reported in 26% and 37% of cases (Von Ah et al. 2017; Ho et al. 2018) and among a population with a prostate cancer diagnosis in the previous 0–23 years (mean 4 years) and partially at work, 10% or 22% reported a reduction of their work ability (Fosså and Dahl 2015). As the studies made use of different ways to assess work ability at various moments after diagnosis and also included different types of cancer, case–control studies offer a possibility to make comparisons between workers with and workers without a past cancer diagnosis. Six studies made use of a reference group or a norm group, mostly beyond the first two years after diagnosis of which five studies found that work ability was lower in workers with a past cancer diagnosis, than in workers without such a diagnosis (Gudbergsson et al. 2008a, 2011; Lee et al. 2008; Lindbohm et al. 2012; Carlsen et al. 2013). Only one study, using a sample 2–6 years after different types of cancer diagnosis, did not report any differences (Taskila et al. 2007). These results demonstrate that work ability tends to be lower among cancer survivors than among samples without a past cancer diagnosis also on the long term. In summary, a number of the cross-sectional and case–control studies showed that workers more than 2 years past cancer diagnoses experience a lower level of work ability than before the cancer diagnosis.

An important finding was that a lower work ability at baseline was one of the strongest predictors of poorer follow-up work ability at 6 months after treatment among a sample with colorectal cancer in one of the longitudinal studies (Bains et al. 2012). Also in a cross-sectional study among a sample 1–16 years after breast cancer diagnosis, the retrospectively self-reported work ability during treatment, as well as that before diagnosis, was associated with current work ability (Cheung et al. 2017). Moreover, in a cross-sectional study 2–6 years after primary treatment of breast, testicular or prostate cancer, mental work ability (and not physical work ability) correlated with lower current work ability (Gudbergsson et al. 2008b). Another finding is that a higher current work ability is associated with work continuation one year later (Duijts et al. 2017).

Furthermore, self-employment among cancer survivors appeared to be a predictor for lower work ability (Torp et al. 2012). Moreover, the negative effect of self-employment on work ability among cancer survivors was reported to be mediated by reduced working hours and a negative cancer-related financial change (Torp et al. 2017). All in all, self-employed, without employees (freelancers) or with employees (entrepreneurs), were not a prominent focus in the included studies. The few available results among the non-salaried show a lower work ability and the importance of negative changes in the financial situation.

Gender differences in work ability among cancer survivors were also reported, but it is difficult to present an overview of possible gender differences with regard to work ability, as factors like type of cancer (and connected gender and age differences) and differences in physical and mental work ability cloud the issue. For instance, breast cancer, testicular cancer and prostate cancer have different profiles with regard to gender and age. Men had a higher current work ability (8.4, SD 1.8) than women (8.0, SD 2.1), *p* < 0.04, effect size = 0.20, while no gender differences were reported for current work ability in the group of matched controls without a past cancer diagnosis (8.6, SD 1.6) (Gudbergsson et al. 2011). Furthermore, female survivors had lower mental work ability than controls (effect size 0.30, *p* < 0.001) but no lower physical work ability, while male survivors had lower physical work ability (effect size 0.37, *p* < 0.001) and also lower mental work ability (effect size 0.27, *p* = 0.004) than male controls (Gudbergsson et al. 2011). In a study among workers 15–39 months after a diagnosis with one of various types of the most common cancer types high current work ability was reported for men (8.6, SD 1.8), as well as for women (8.6, SD 1.7) (Torp et al. 2012). Taskila et al. (2007) reported the highest mean current work ability for testicular cancer (9.0) and the lowest for prostate cancer (8.0), in a study which also covered breast cancer and lymphoma. Furthermore, in another study no difference in work ability between men with testicular cancer diagnosis (8.8) and controls (8.7) was reported, while prostate cancer survivors had a lower work ability (8.3) than controls (*p* < 0.01) (Lindbohm et al. 2012).

## Results: late effects of cancer treatment and work ability

### Physical complaints and work ability

Eight (22%) of the included studies analyzed a possible association between late physical complaints and work ability. One study had a case–control design (Gudbergsson et al. 2011), and the other studies were cross-sectional (Gudbergsson et al. 2008b; Moskowitz et al. 2014; Fosså and Dahl 2015; Dahl et al. 2016, 2019; Torp et al. 2017; Ho et al. 2018). In the studies physical impairments or the experienced limitations were associated with lower work ability or were seen more frequently in cases of suboptimal work ability beyond 2 years after diagnosis. In short, physical complaints after cancer treatment continue to show associations with lower work ability beyond the first two years after cancer diagnosis.

### Fatigue and work ability

Four (11%) of the included studies analyzed a possible association between late fatigue and work ability. Carlsen et al. (2013), used the first WAI item in a case–control study design, and reported that fatigue was associated with reduced current work ability 5–8 years after a breast cancer diagnosis, and that this association was stronger among cancer survivors (OR 10.7, CI 3.31–34.3) than among the controls (OR 4.11, CI 1.97–8.57), suggesting moderation. The other three studies were cross-sectional. In one of these studies the complete WAI to assess work ability was used, and general, physical, and mental fatigue were reported to be less common in breast cancer survivors with optimal work ability. A higher level of physical fatigue was significantly associated with poorer work ability (Ho et al. 2018). Another cross-sectional study used the first item of the WAI to assess work ability and reported that those with low work ability had significantly higher mean levels of total fatigue (Dahl et al. 2019). Another cross-sectional study did not report a significant association of fatigue with work ability, however fatigue was part of more comprehensive constructs, making specific inferences difficult. Furthermore, in this study work ability was assessed by a multiple choice question regarding being unable to work full time, unable to work the same as before cancer or unable to work at all (Moskowitz et al. 2014) To summarize, the scarce data demonstrate that fatigue can be associated with lower work ability among workers with a past cancer diagnosis.

### Cognitive complaints and work ability

Four (11%) of the included studies analyzed a possible association between late cognitive complaints and work ability. The study designs were all cross-sectional. In this systematic literature review attentional fatigue, i.e. experiencing lower levels of attention, is regarded as a cognitive complaint. A significant relationship (*β* = 0.627, *p* < 0.001) between higher levels of attention and perceived work ability assessed by the complete WAI, was reported by Von Ah et al. (Von Ah et al. 2017). Attentional fatigue explained 40% of the variance in perceived work ability among 68 breast cancer survivors on average 5 years after diagnosis. Von Ah et al. (2018) also reported that cognitive impairment was associated with poorer work ability (*β* =  − 0.66, *p* < 0.000) and that perceived cognitive ability was significantly related to higher levels of work ability (*β* = 0.47, *p* < 0.000). Furthermore, Ho et al. (2018) reported breast cancer survivors (3–8 years after diagnosis) to have lower scores for cognitive functioning in case of suboptimal work ability. Another study, by Moskowitz et al. (2014), also included cognitive symptoms, but as part of more comprehensive constructs, making specific inferences difficult. So, although results are scarce, recent studies indicate that cognitive complaints can be associated with low work ability among working cancer survivors.

## Results: current job resources and work ability

As has already been stated, job resources can be of importance for work functioning, also among workers who returned to work after cancer treatment and experiencing any late effects of cancer treatments. Job resources can among others be provided by (1) social support, (2) autonomy, (3) leadership style, (4) coaching, or (5) organizational culture (Demerouti et al. 2001). Of these job resources the current experienced level and their possible association with work ability was taken into consideration in nine (25%) of the included studies; three case–control studies and six cross-sectional studies.

Social support by colleagues was reported to be associated with positive outcomes with regard to higher work ability in case–control studies (Taskila et al. 2007; Lindbohm et al. 2012; Carlsen et al. 2013), as well in cross-sectional studies (Gudbergsson et al. 2008b; Torp et al. 2012; Musti et al. 2018). For instance, a high level of cancer-related support by colleagues was associated with higher work ability 15–39 months after diagnosis, also in multivariate regression (Torp et al. 2012). Social support by supervisors was reported to be associated with positive outcomes with regard to higher work ability as well in case–control studies (Lindbohm et al. 2012; Carlsen et al. 2013), as in cross-sectional studies (Torp et al. 2012; Musti et al. 2018). For instance, less help and support from a supervisor was significantly associated with reduced work ability among workers 5–8 years after breast cancer diagnosis (Carlsen et al. 2013).

Three cross-sectional studies analyzed a possible association of autonomy at work with work ability, although the construct of autonomy was defined somewhat differently. In two of these studies the respondents reported the ‘decision latitude’ (opportunities to learn new things at work and decide how to carry out the work tasks) at the time of the cancer diagnosis (Torp et al. 2012, 2017). Decision latitude was found to be significantly related with work ability among a sample workers who returned to work after various cancer diagnoses, 6% of whom were self-employed (Torp et al. 2012). In addition, it was also reported that the self-employed experienced a higher decision latitude, preventing low work ability (Torp et al. 2017). Furthermore, Cheung, Ching, Chan, Cheung, and Cheung (2017) reported ‘control’, a related concept, to be correlated with work ability (Rs = 0.29, *p* = 0.04).

Leadership style, coaching and organizational culture were assessed in almost none of the included studies. However, social climate at work, a concept related to organizational culture (Ehrhart and Schneider 2016), was assessed in two studies (Taskila et al. 2007; Lindbohm et al. 2012), with only one study analyzing a possible association with work ability. This study showed that a better social climate at work was related to a higher mental work ability (Taskila et al. 2007). The only behavior of supervisors related to leadership style that was assessed in some of the studies was social support from supervisors and their avoidance behavior. Worth noting is that male workers with a cancer diagnosis experienced lower work ability as a result of supervisors’ avoidance behavior (*p* < 0.001), while female workers with a cancer diagnosis in their past experienced lower work ability if avoidance behavior of colleagues was higher (*p* < 0.001) (Lindbohm et al. 2012).

All in all, the attention paid to job resources among the included studies was limited. Nevertheless, the scarce results indicate a positive association between job resources and work ability, although no data on job resources that affect the strength of the association of the late effects with work ability have been found.

## Discussion

As high numbers of working people diagnosed with cancer re-enter the workplace and the group of workers with a cancer diagnosis in their life history will continue to expand, it is important to have an overview over the current state of knowledge about the course of work ability after diagnosis, and about the associations between late effects of cancer treatment and work ability. Knowledge about the role of job resources (social support, autonomy, leadership style, coaching, and organizational culture) in this is also relevant.

The searches included 2303 records in total, and 36 studies were selected. A quality assessment was used to clarify the quality across studies and we found that most research was cross-sectional (50%). These studies and the six case–control studies were mostly completely or in part focused on workers beyond two years past cancer diagnosis. However, only two of the 12 cohort studies had a follow-up beyond 2 years after diagnosis. 

It is an important finding that studies with various study populations and study designs demonstrate that work ability seems to be lowered shortly after the start of cancer treatment and tends to recover during the first two years after the diagnosis, although work ability might still be lower than in healthy populations. Because there is a lack of longitudinal data beyond the first two years after diagnoses, the further course of work ability is not clear. Differences in the level of work ability between workers with different types of cancer diagnosis in the past are reported. Late physical complaints, fatigue or cognitive complaints are associated with lower work ability across all relevant studies. None of these studies had a longitudinal design.

Social support and characteristics of autonomy were assessed in some of the studies, indicating that these current job resources are associated with higher work ability, in line with results in the healthy population (Gould et al. 2000) and also in populations experiencing chronic health problems (Leijten et al. 2014). No data were available on the possible buffering effects of social support and autonomy on the relationship between late effects of cancer treatments and work ability. Organizational culture in general was not investigated, only social climate at work in one study, which was positively related to a higher work ability. No results were found for leadership style, and coaching. In short, research on late effects of cancer treatment and work ability among workers past cancer diagnosis has not yet been enriched or combined with investigations of possible buffering by job resources.

### Limitations

First, of the 36 studies included, ten studies (28%) solely concerned workers with a breast cancer diagnosis, which may have caused bias. The other studies used in this review included considerable variations in type(s) of cancer and cancer treatments. However, the impact of differences in diagnosis is not clear. For instance, survivors of testicular cancer reported the highest work ability (even comparable to controls), survivors with prostate cancer the lowest level, and the breast cancer population in between (Taskila et al. 2007; Lindbohm et al. 2012). It is important to be aware of the very different profiles with regard to gender and age of these types of cancer. Among healthy populations age is generally associated with work ability, younger workers usually estimating their work ability at a higher level (Gould et al. 2000; Berg van de et al. 2010; Bender et al. 2015). Also, variation among participants in the disease status may cause a lack of comparability, as there are differences between studies with regard to including participants with recurrence, or distant metastasis, while awareness of disease progression or the possibility of the cancer not being curable, might influence perceived work ability.

Second, the way that work ability was measured did not seem to influence the results. The complete WAI (Work Ability Index) was used in a few studies only, while the vast majority of studies used only one or more of the items adopted from the WAI, with the first item (current work ability compared to life-time best) being used most frequently. The complete WAI is reported to be a very predictive and cross-nationally stable instrument (Radkiewicz and Widerszal-Bazyl 2005) to predict work disability, retirement and mortality in a reliable way (Ilmarinen and Tuomi 2004). Furthermore, the first item of the WAI is reported to have a very strong association with the complete WAI (Ahlstrom et al. 2010), and to show similar strong predictive value for the degree of sick leave, health-related quality of life (Ahlstrom et al. 2010) and future disability (Alavinia et al. 2009). Although in the general populations the use of the complete WAI might result in a higher probability of lower work ability in women compared to using only the first item of the WAI (El Fassi et al. 2013), using only one item of the WAI is regarded as a good alternative for the complete WAI. A minority of the included studies did not use any of the WAI items, but used different surveys, ad hoc questions, a perception of the participant, etcetera. In short, when interpreting results on work ability in workers with a past cancer diagnosis, conscientiousness in reviewing the assessment tool of work ability is wise, although the results across the studies included in this review do not lead to different conclusions.

Third, the late effects of cancer treatment evaluated in this systematic literature review were not all possible prevalent late effects. For instance, depression was not included, and the effect of co-morbidities was not clear. However, the scarce studies that investigated a possible association of late physical complaints, fatigue and cognitive complaints with work ability, indicated that these complaints after cancer treatment were associated with lower work ability in almost all included studies. It is important to be alert of the likelihood of stronger associations of specific complaints with work ability in the cancer population, as this was already reported for fatigue in one of the included studies (Carlsen et al. 2013). More knowledge is needed to be able to know what subgroups are at risk and aim rehabilitation interventions at the right objectives. Furthermore, it is important to realize that the prevalence of late effects might also differ due to different types of treatment (Stein et al. 2008), while these differences are not always taken into account.

Fourth, the work status, the type of employment and the personal work histories of the study participants were not clear in a vast majority of the studies. Study samples did not in all instances include participants who had fully recovered 100% of their previous working hours currently or were not always entirely actively at work during the study’s data selection for unknown reasons. Only some studies mentioned type of work, like blue or white collar. Also, information on previous work adjustments, previous changes of job or of employer, was mostly not presented. So, results might be biased by those not actually active in work, by differences in type of work or already made adjustments in job demands made in an earlier stage. Furthermore, the setting of 75% of the studies was a European country, preventing global generalizability.

Fifth, only 13 (36%) of the 36 studies mentioned the inclusion of self-employed workers; freelancers, or entrepreneurs (Taskila and Lindbohm 2007; Gudbergsson et al. 2008a; Lee et al. 2008; De Boer et al. 2011; Torp et al. 2017, 2012; Moskowitz et al. 2014; Von Ah et al. 2017; Cheung et al. 2017; Hartung et al. 2018; Ortega et al. 2018; Wolvers et al. 2019; Tamminga et al. 2019). However, the self-employed might have different characteristics in regard to age, educational level, and gender and decision latitude, as was reported in one of the studies (Torp et al. 2017). Also, a recent European multi-country study (Torp et al. 2018), reported that differences in work ability could be observed between salaried and self-employed but that the direction and magnitude of these differences differed across countries. The variation between different kinds of self-employment should probably be considered too, as self-employment occurs in very different professional areas, and among the healthy population agricultural entrepreneurs, for instance, have a lower work ability than other occupational groups (Gould et al. 2000). The conclusion from this review is that the non-salaried workers among cancer survivors are reported to have a lower work ability than salaried workers. However, differentiation in occupational groups within the self-employed is not clear, stressing the need to take this into account as self-employment shows varying profiles. This review does not clarify whether predictors of lower work ability in this type of employment differ from the predictors of lower work ability in the salaried work situation. Nevertheless, the role of reduced working hours and a negative cancer-related financial change underlines that targets for occupational rehabilitation in this group of workers could also be interventions directed at business support, as some rehabilitation providers focusing on the self-employed are already offering. Future studies should focus on the needs of this specific group of the non-salaried workers with a past cancer diagnosis.

Finally, this review was limited to five well-known job-resources for the general working population. Other job resources, such as growth opportunities, performance feedback or organizational prestige, might also be relevant for the salaried, and also or even exclusively for the non-salaried. Furthermore, also personal resources are important (McGonagle et al. 2015), however these were not the focus of this review.

### Strengths

This is the first review to focus on late effects of cancer treatment, work ability and job resources. This review combines findings on the effects of cancer treatment with work ability (Ilmarinen et al. 2005), and with the Job Demands-Resources (JD-R) model (Demerouti et al. 2001), which is unique to our knowledge. The goal of sustainable work participation of cancer survivors needs tailored interventions (De Boer et al. 2020b) and the outcome measure of work ability is an important factor in this research area. This review integrates concepts originated in different research disciplines with the intention to be able to focus on targets in the workplace to preserve and enhance work ability among workers experiencing late effects of cancer treatment beyond the first two years after cancer diagnosis.

## Conclusion

To conclude, this systematic literature review confirms that a lowered work ability after the start of cancer treatment, might recover during the first two years after diagnosis. However, at two or more years beyond cancer diagnosis work ability might still be lower than before the cancer diagnosis. The course of work ability among workers beyond the first two years after diagnoses is unknown as no longitudinal data are available. Longitudinal research in salaried and non-salaried populations is needed to study in more detail what factors are important for sustainable occupational rehabilitation after cancer treatment. Besides this, an interesting methodological finding is that although the majority of the studies uses one of more items of the Work Ability Index (WAI) to assess work ability, also a substantive part of the included studies makes use of a variety of validated and non-validated measurement tools. The method to measure work ability did not seem to lead to different conclusions.

Physical complaints, fatigue and cognitive complaints may be present as late effects of cancer treatment beyond two years after diagnosis and can be associated with a lower level of work ability. However, data on the association between late effects and work ability is scarce. Furthermore, it is unknown if late effects of cancer treatment diminish work ability beyond two years after being diagnosed with cancer because longitudinal studies are lacking.

Furthermore, this review also makes clear that the job resources leadership style, coaching and organizational culture were not taken into account in studies on late effects of cancer treatment and work ability, and that for the job resources that were included (autonomy and social support in the workplace) no possible buffering effect was analyzed. However, autonomy and social support were associated with higher work ability and therefore are important for work functioning among workers past cancer diagnosis and it is recommended to enhance these job resources as much as possible.

This review indicates that there is an urgent need to close this gap in our knowledge. It is important to study late effects of cancer treatment, work ability and job resources in combination within studies among various samples of workers with a past cancer diagnosis, as well in large international cohorts. These studies need to be carried out beyond the first two years of cancer diagnosis. A focus on a broad range of job resources is essential, both for salaried and self-employed workers. It should be clear what range of job resources might accelerate a recovery of work ability, creating an important step towards clarifying the issue of the rehabilitation of work ability beyond return to work among workers with a history of cancer.

## Electronic supplementary material

Below is the link to the electronic supplementary material.

Supplementary file1 (DOCX 16 kb)
