# Supplementary material for: The associations between late effects of cancer treatment, work ability and job resources: a systematic review
Source: Int Arch Occup Environ Health. 2020 Sep 15;94(2):147–89. doi: 10.1007/s00420-020-01567-w (PMC7873002; doi:10.1007/s00420-020-01567-w)
Supplement: Supplementary file 1 — Supplementary file1 (DOCX 16 kb) [file 420_2020_1567_MOESM1_ESM.docx]

**Appendix 1. Search strategies**

The searches below yielded the following results on March, 2020; PsycINFO (164 references), MEDLINE (811 references), Business Source Premier (47 references), ABI/Inform (91 references), CINAHL- NOT Medline (99 references), Cochrane Library (68 references), Web of Science (1.021 references). This yielded a total of 2.301 references.

**PsycINFO (Ovid, 1806 to 10^th^ March 2020)**

1. neoplasms/ OR breast neoplasms/ OR endocrine neoplasms/ OR leukemias/ OR melanoma/ OR (cancer* OR tumo?r* OR neoplasm* OR carcinoma* OR melanom* OR leukemi* OR melanom*).ti,ab,id.
2. (((work OR employ*) ADJ2 (ability OR continu* OR sustain*)) OR occupationally active OR beyond return to work* OR working patient*).ti,ab,id,tm.
3. 1 AND 2

Key: / = subject heading, ti = title, ab = abstract, id = key concepts (other keywords added by PsycINFO indexers to supplement the subject headings), tm = tests & measures

**MEDLINE (Ovid MEDLINE, including epub ahead of print, in-process & other non-indexed citations and Ovid MEDLINE Daily, 1946 to 10^th^ March 2020)**

1. exp neoplasms/ OR (cancer* OR tumo?r* OR neoplasm* OR carcinoma* OR melanom* OR leukemi* OR melanom* OR lymphoma*).ti,ab,kf.
2. (((work OR employ*) ADJ2 (ability OR continu* OR sustain*)) OR occupationally active OR beyond return to work*).ti,ab,kf.
3. 1 AND 2

Key: / = medical subject heading (MeSH), ti = title, ab = abstract, kf = author supplied keywords

**Business Source Premier (EBSCO, 1886 to 10^th^ March 2020)**

1. TI("cancer*" OR "tumo#r*" OR "neoplasm*" OR "carcinoma*" OR "melanom*" OR "leukemi*" OR "melanom*") OR AB("cancer*" OR "tumo#r*" OR "neoplasm*" OR "carcinoma*" OR "melanom*" OR "leukemi*" OR "melanom*") OR KW("cancer*" OR "tumo#r*" OR "neoplasm*" OR "carcinoma*" OR "melanom*" OR "leukemi*" OR "melanom*")
2. TI((("work" OR "employ*") N2 ("ability" OR "continu*" OR "sustain*")) OR "occupationally active" OR "beyond return to work*") OR AB("work ability" OR (("work" OR "employ*") N2 ("continu*" OR "sustain*")) OR "occupationally active" OR "beyond return to work*") OR KW("work ability" OR (("work" OR "employ*") N2 ("continu*" OR "sustain*")) OR "occupationally active" OR "beyond return to work*")
3. 1 AND 2

Key: TI = title, AB = abstract, KW = author supplied keywords

**CINAHL (EBSCO, CINAHL Plus with full text, 1937 to 10^th^ March 2020)**

1. MH("Neoplasms+" OR "cancer patients" OR "cancer survivors") OR TI("cancer*" OR "tumo#r*" OR "neoplasm*" OR "carcinoma*" OR "melanom*" OR "leukemi*" OR "melanom*") OR AB("cancer*" OR "tumo#r*" OR "neoplasm*" OR "carcinoma*" OR "melanom*" OR "leukemi*" OR "melanom*") OR KW("cancer*" OR "tumo#r*" OR "neoplasm*" OR "carcinoma*" OR "melanom*" OR "leukemi*" OR "melanom*")
2. TI((("work" OR "employ*") N2 ("ability" OR "continu*" OR "sustain*")) OR "occupationally active" OR "beyond return to work*") OR AB("work ability" OR (("work" OR "employ*") N2 ("continu*" OR "sustain*")) OR "occupationally active" OR "beyond return to work*") OR KW("work ability" OR (("work" OR "employ*") N2 ("continu*" OR "sustain*")) OR "occupationally active" OR "beyond return to work*")
3. 1 AND 2
4. 3 NOT MEDLINE

Key: MH = CINAHL Subject Headings, TI = title, AB = abstract, KW = author supplied keywords

**Web of Science (Web of Science Core Collection, 1975 to 13^th^ March 2020)**

1. TS=("cancer*" OR "tumor*" OR "tumour*" OR "neoplasm*" OR "carcinoma*" OR "melanom*" OR "leukemi*" OR "melanom*")
2. TS=((("work" OR "employ*") NEAR/2 ("ability" OR "continu*" OR "sustain*")) OR "occupationally active" OR "beyond return to work*")
3. 1 AND 2

Key: TS = topic, which includes title, abstract, author keywords and Web of Science Keywords Plus

**ABI/Inform (Proquest, 1971 to 13^th^ March 2020)**

1. TI,AB,SU("cancer*" OR "tumor*" OR "tumour*" OR "neoplasm*" OR "carcinoma*" OR "melanom*" OR "leukemi*" OR "melanom*")
2. TI,AB,SU((("work" OR "employ*") NEAR/2 ("ability" OR "continu*" OR "sustain*")) OR "occupationally active" OR "beyond return to work*")
3. 1 AND 2
4. From 3 exclude wire feeds, trade journals and newspapers

Key: TI = title, AB = abstract, SU = subject headings

**Cochrane Library to 13^th^ March 2020)**

1. "cancer*":ti,ab,kw OR "tumor*":ti,ab,kw OR "tumour*":ti,ab,kw OR "neoplasm*":ti,ab,kw OR "carcinoma*":ti,ab,kw OR "melanom*":ti,ab,kw OR "leukemi*":ti,ab,kw OR "melanom*":ti,ab,kw
2. (("work":ti,ab,kw OR "employ*":ti,ab,kw) NEAR/2 ("ability":ti,ab,kw OR "continu*":ti,ab,kw OR "sustain*":ti,ab,kw)) OR "occupationally active":ti,ab,kw OR "beyond return to work*":ti,ab,kw
3. 1 AND 2

Key: ti = title, ab = abstract, kw = keyword
